# Rectal Tumor Stiffness Quantified by *In Vivo* Tomoelastography and Collagen Content Estimated by Histopathology Predict Tumor Aggressiveness

**DOI:** 10.3389/fonc.2021.701336

**Published:** 2021-08-13

**Authors:** Jiaxi Hu, Jing Guo, Yigang Pei, Ping Hu, Mengsi Li, Ingolf Sack, Wenzheng Li

**Affiliations:** ^1^Department of Radiology, Xiangya Hospital, Central South University, Changsha, China; ^2^Department of Radiology, Charité – Universitätsmedizin Berlin, Berlin, Germany

**Keywords:** tomoelastography, rectal cancer, collagen content, tumor aggressiveness, multifrequency magnetic resonance elastography, stiffness, risk factors

## Abstract

**Purpose:**

To investigate the significance of collagen in predicting the aggressiveness of rectal tumors in patients, examined *in vivo* based on tomoelastography quantified stiffness and *ex vivo* by histologically measured collagen volume fraction (CVF).

**Experimental Design:**

170 patients with suspected rectal cancer were prospectively enrolled and underwent preoperative magnetic resonance imaging (MRI) and rectal tomoelastography, a technique based on multifrequency magnetic resonance elastography. Histopathologic analysis identified eighty patients with rectal cancer who were divided into subgroups by tumor-node (TN) stage, prognostic stage, and risk level. Rectal tumor stiffness was correlated with histopathologic CVF. Area-under-the-curve (AUC) and contingency analysis were used to evaluate the performance of rectal stiffness in distinguishing tumor stages which was compared to standard clinical MRI

**Results:**

*In vivo* tomoelastography revealed that rectal tumor stiffened significantly with increased TN stage (p<0.05). Tumors with poorly differentiated status, perineural and lymphovascular invasion also displayed higher stiffness than well-to-moderately differentiated, noninvasive tumors (all p<0.05). Similar to *in vivo* stiffness, CVF indicated an abnormally high collagen content in tumors with perineural invasion and poor differentiation status. CVF was also positively correlated with stiffness (p<0.05). Most importantly, both stiffness (AUROC: 0.82) and CVF (AUROC: 0.89) demonstrated very good diagnostic accuracy in detecting rectal tumors that have high risk for progressing to an aggressive state with poorer prognosis.

**Conclusion:**

In human rectal carcinomas, overexpression of collagen is correlated with increased tissue stiffness and high risk for tumor advancing more aggressively. *In vivo* tomoelastography quantifies rectal tumor stiffness which improves the diagnostic performance of standard MRI in the assessment of lymph nodes metastasis. Therefore, *in vivo* stiffness mapping by tomoelastography can predict rectal tumor aggressiveness and add diagnostic value to MRI.

## Introduction

Colorectal cancer (CRC) is the third most common cancer in men and the second most common cancer in women (1). The Union for International Cancer Control and American Joint Committee on Cancer (AJCC) tumor-node-metastasis (TNM) staging system is widely used for the clinical assessment of patients with colorectal cancer (2). The TNM system has been updated and refined over the years by incorporating new risk factors and introducing finer subcategories to improve its accuracy and robustness (3). As recommended by the National Comprehensive Cancer Network (NCCN), histopathologic features such as number of positive nodes, lymphovascular invasion (LVI), perineural invasion (PNI), and poor differentiation have been recognized as high-risk factors for local recurrence and distant metastasis (4). Moreover, observations of increased collagen crosslinking and linearization in human CRC samples (5, 6) contributed to the recognition that the amount, composition and structure of extracellular matrix (ECM) in the tumor microenvironment promotes CRC progression (7–11).

Altered collagen content and alignment translates to macroscopic changes in biomechanical tissue properties that can be non-invasively quantified *in vivo* by magnetic resonance elastography (MRE) (12). As demonstrated by extensive literature data, MRE uniquely provides parameters of viscoelasticity that are sensitive to the amount and structure of collagen networks (13–15). The diagnostic power of MRE has been demonstrated in patients with tumors in the liver (16, 17), breast (18, 19), kidney (20), brain (21–23), prostate (15, 24) and pancreas (25–27). To date, MRE has never been applied to patients with CRC and hence stiffness has not yet been used as a diagnostic parameter for assessing CRC. Magnetic resonance imaging (MRI) based on the enhancement of contrast agents and magnetic relaxation times depicts tumor morphology and is recommended as key modality for the noninvasive staging of rectal tumors by international guidelines (28–30). However, morphological features provided by routine MRI are limited in assessing lymph nodes status (31–33), histopathologic risk factors such as PNI, LVI, as well as the degree of tumor differentiation. MRE could be of complementary value to current MRI by providing stiffness as a quantitative imaging marker for ECM remodeling during tumor progression for improved preoperative staging, risk stratification, and prediction of therapeutic efficiency in rectal cancer.

The general feasibility of MRE in colorectal cancer has been demonstrated in a mouse model (34); however, clinical rectal MRE has been compromised by introducing shear waves into the gastrointestinal tract and generating consistent stiffness maps of this body region. We here overcome these challenges by employing a novel tomoelastography technique that includes multiple actuators operated by compressed air, multifrequency MRE, and noise-robust data processing (35, 36).

We hypothesize that tomoelastography-measured rectal stiffness may discriminate patients with different prognostic stages of rectal cancer. Our study has four objectives: 1) to demonstrate the feasibility and reproducibility of rectal MRE based on tomoelastography in healthy volunteers and patients; 2) to quantify for the first time values of rectal tumor stiffness for clinical diagnosis; 3) to investigate the correlation between histopathologically measured collagen content with tumor stiffness; and 4) to analyze if tomoelastography adds diagnostic value to standard clinical MRI using histopathology as reference standard.

## Material and Methods

### Study Design and Participants

The institutional review board approved our prospective study (No.201903078), and all participants gave written informed consent.

Twelve healthy volunteers (median age, 25 years; range, 23-54 years; 4 females; BMI, 20.8 ± 2.6), and 170 patients (median age, 56 years; range, 22-82 years; 65 females; BMI, 22.6 ± 4.4) with suspected rectal cancer were recruited from Nov. 2018 to Dec. 2019.

To test the feasibility and reproducibility of colorectal tomoelastography, all volunteers were investigated twice, separated by 35 ± 5 days.

All 170 patients underwent routine clinical rectal MRI and tomoelastography. Exclusion criteria were: 1) adjuvant treatment between MRI and surgery (n=53); 2) time between MRI and surgery ≥2weeks (n=12); 3) transfer to other hospitals for further treatment (n=10); 4) endoscopic submucosal dissection instead of radical surgery (n=6); 5) histopathologically proven nonrectal adenocarcinoma (n=7); and 6) poor image quality due to severe peristaltic artifacts (n=2). We finally included 80 patients with histopathologically proven rectal adenocarcinoma in surgical specimens. **Supplementary Figure 1** provides a flowchart of patient recruitment and selection criteria for MRI and tomoelastography.

### Image Acquisition

All patients started a fluid diet one day before MRI and followed a strict 4-hour fasting regimen prior to imaging. MRI was performed at 3T (Magnetom Prisma, Siemens Healthcare, Germany) with an 18-channel phased-array body coil. Routine rectal T2-weighted (T2w) images with 3×3 mm^2^ in-plane resolution were acquired with a 2D fast-spin-echo (FSE) sequence in oblique axial, sagittal, and coronal planes. Additionally, 3D FSE (SPACE) T2w images with 0.8×0.8mm^2^ in-plane resolution were obtained. Total acquisition time for the anatomical images was 12 min.

Rectal tomoelastography was performed using a similar sequence and setup as described in (35). Briefly, mechanical waves of vibration frequencies of 40, 50, 60, and 70 Hz were transferred to the pelvic region by three surface-based, pressurized-air-driven actuators – two placed posterior (0.8 bar static pressure) and one anterior to the pelvis, i.e., on top of the pubic symphysis (0.7 bar static pressure). The complete 3D wave field was acquired using a single-shot, spin-echo echo-planar-imaging (SE-EPI) sequence with flow-compensated motion-encoding gradient (MEG). The full vibration period was sampled at eight phase offsets. Fifteen consecutive 5-mm-thick sagittal slices with 3×3 mm^2^ resolution were acquired during free breathing. MRE frequencies were set to 47.89, 47.89, 47.89 and 52.41 Hz which were optimized for the vibration frequencies of 40, 50, 60 Hz and 70 Hz, correspondingly. Further imaging parameters were: echo time=56ms; repetition time=1670ms; parallel imaging with GRAPPA factor 2; and MEG amplitude of 50mT/m. Total acquisition time was 3.5 min.

### Image Analysis

A radiologist with 5 years of experience in gastrointestinal imaging assessed tumor location, TNM stage, circumferential resection margin (CRM) involvement, and extramural vascular invasion (EMVI) on T2w images using the DISTANCE method (37). DISTANCE is a systematic approach for an adequate assessment of all clinically relevant features based on MR images. It is essential for treatment decision making. In DISTANCE, DIS stands for the distance from the inferior part of the tumor to the transitional skin; T is for T staging, A is for Anal complex, N is for Nodal staging, C refers to Circumferential resection margin, and E stands for Extramural vascular invasion. Using DISTNACE approach, MRI based T and N staging were assigned to each patient.

MRE datasets were processed using wave-number multifrequency-inversion (k-MDEV) (36) to generate parameter maps of shear wave speed *c* (in m/s). Being recovered from the real part of complex wave numbers, *c* is considered a surrogate parameter of stiffness. We use *c* when providing quantitative information and the term “stiffness” when discussing qualitative changes in *c*. Data processing was performed using the *k*-MDEV pipeline available at www.bioqic-apps.com. For tumor characterization, 9 to 18 circular regions of interest (ROIs) measuring 0.3 ± 0.02 cm^2^ were placed in the anterior and/or posterior rectal wall in 3 consecutive slices of covering the largest solid tumor cross-section with reference to anatomical T2w images, avoiding necrosis, cyanosis, and blood vessels. Stiffness values were averaged within these manually defined ROIs. Distal tumor-adjacent tissue (DTT) 2 cm away from the tumor was analyzed in 6 circular ROIs measuring 0.1 ± 0.02 cm^2^ as reference. For healthy rectal wall assessment in volunteers, ROIs identical to those used for DTT in patients were placed in both the anterior and posterior wall in 3 consecutive slices. A radiologist blinded to clinical outcome placed all ROIs using both MRE magnitude images and the corresponding elastograms.

### Histopathologic Analysis

Tumor tissue samples from 80 patients were firstly stained with hematoxylin and eosin (H&E). Based on H&E staining, routine histopathologic reports of resected specimens provided TN stages, tumor differentiation, PNI, and LVI. Overall tumor differentiation was categorized as well to moderate *vs*. poor (≥50 *vs*. <50% glandular area) using the WHO classification system (38). Staging was done by two pathologists specializing in digestive tract tumors using the TNM classification system (8^th^ edition) recommended by the American Joint Committee on Cancer (AJCC).

To visualize and quantify collagen content, Masson’s trichrome staining was additionally performed in tissue sections from 69 patients (11 cases were not stained due to insufficient tissue after H&E) according to protocol described in (39). The sections were scanned using KFBIO KF-PRO-005 EX Digital Imaging System (Ningbo Konfoong Bioinformation Tech Co., Ltd. China) and imaged using a Zeiss microscope. The quantification of histologic fibrosis was performed in three representative fields at 200x magnification with ImageJ software (NIH, USA, http://rsb.info.nih.gov/ij) and expressed as collagen volume fraction (CVF). Color deconvolution was applied to the images using Masson Trichrome vector derived from a color-based calculation algorithm within ImageJ software (40). After deconvolution, the area with green pixels which represent collagen fibers was analyzed and recorded for each image. Finally, CVF was calculated as the ratio between the area with green pixels and the total area of the original, non-deconvoluted image. Analysis of the Masson’s trichrome stained images was performed with the examiner blinded to the clinical histopathologic findings.

### Statistical Analysis

Group means and standard deviations were calculated for different patient groups. Normal distribution was tested with the Shapiro-Wilk test. Significant differences between groups were identified using the unpaired t-test (groups with normal distribution) or Mann-Whitney test (nonnormal distribution). Kruskal-Wallis test was used for 3-group comparison. Categorical variables were analyzed using a chi-square test.

For reproducibility analysis in healthy volunteers, coefficient of repeatability (CR), intraclass correlation coefficient (ICC), and relative absolute difference (RADi) were calculated. Interobserver agreement was evaluated using the ICC along with its 95% confidence interval (CI).

Area-under-the-curve (AUC) and contingency analysis were used to assess diagnostic accuracy in distinguishing tumor stages. The diagnostic performance of combined biomarkers was established using logistic regression analysis. Correlation analysis was performed between *in vivo* rectal tumor stiffness quantified by tomoelastography and the amount of collagen calculated as CVF based on histopathologic staining. Correlation was analyzed by Spearman (nonnormal distribution, categorical variables) and Pearson correlation (normal distribution, continuous variables). To assess the predictive accuracy of tomoelastography and routine MRI for tumor staging, contingency analysis was performed using histopathology as reference standard. For the contingency analysis, as shear wave speed *c* obtained from tomoelastography is a continuous variable, it was dichotomized with the corresponding cutoffs from the AUC analysis for different cancer staging. Statistical analysis was performed using SPSS (version 22.0; IBM, Armonk, NY). P-values <.05 were considered statistically significant.

## Results

### Clinicopathologic Characteristics

Based on histopathological analysis of surgically resected specimens, 32 patients whose tumors did not extend beyond the rectal muscularis propria were grouped and assigned to pT1–2 stages (16 pT1 cases and 16 pT2 cases), while the remaining 48 patients with confirmed tumor infiltration beyond the muscularis propria were pooled into pT3–4 stages (41 pT3 cases and 7 pT4 cases). In terms of lymph node involvement, 54 patients were free of lymph node metastasis (pN0) whereas 26 patients had different degrees of lymph node metastasis (21 pN1 cases and 5 pN2 cases) were pooled into the pN1–2 group. The prefix p in the stages represents pathology.

Furthermore, according to the 8^th^ version of the TNM classification system recommended by the AJCC (2), patients were assigned to three different pathology-based prognostic stages (progStages): 0-I (n=26), II (n=27), and III-IV (n=27), based on a collective consideration of their individual T, N, and M stages. Additionally, patients were divided into a high-risk and a low-risk group for local tumor recurrence and aggressive progression, according to the management strategies of rectal cancer in Europe (5). The risk of cancer progression and prognosis were assessed in order to make appropriate treatment decisions. Patients with lymph node involvement, positive LVI and PNI status, and poorly differentiated tumors were assigned to the high-risk group (n=36), and the remaining patients (n=44) were classified as low-risk. The clinicopathologic features, routine MRI findings, and tomoelastography parameters of the total population and subgroups are presented in **Table 1**.

**Table 1:** Clinical and pathologic characteristics of the patient population and subgroups.

	All patients (n=80)	High-risk group (n=36)	Low-risk group (n=44)	*P* value (low- *vs* high-risk)
**Patient characteristics**				
Age (year)	57.8 ± 11.2	57.3 ± 13.6	58.2 ± 9.2	0.73
Sex (M/F)	44/36	19/17	25/19	0.72
BMI (kg/m²)	21.9 ± 4.1	21.1 ± 4.6	22.5 ± 3.5	0.14
CEA (ug/L)	4.6 ± 1.2	6.0 ± 15.1	3.5 ± 5.1	0.32
**MRI features**				
mT (T1/T2/T3/T4)	13/18/38/11	1/5/26/4	12/13/17/2	<0.001
mN (N0/N1/N2)	37/31/12	9/17/10	28/14/2	<0.001
mM (M0/M1)	79/1	35/1	44/0	–
CRM (-/+)	74/6	31/5	43/1	<0.05
EMVI (-/+)	67/13	26/10	41/3	0.012
Thickness (mm)	14.7 ± 7.8	13.8 ± 6.3	15.4 ± 8.8	0.377
Length (mm)	41.0 ± 16.8	44.9 ± 16.0	37.8 ± 17.0	0.060
Hyperintensity on T2w images (-/+)	67/13	25/11	42/2	0.002
**Histopathologic features**				
pT (Tis/T2/T3/T4)	16/16/41/7	1/5/23/7	15/11/18/0	–
pN (N0/N1/N2)	54/21/5	10/21/5	44/0/0	–
LVI (-/+)	68/12	24/12	44/0	–
PNI (-/+)	72/8	28/8	44/0	–
Mucinous differentiation (-/+)	74/6	30/6	44/0	<0.001
Tumor differentiation (well-to-moderate *vs* poor)	71/9	36/9	44/0	–

CEA, carcinoembryonic antigen; T, tumor; N, node; M, metastasis; CRM, circumferential resection margin; EMVI, extramural vascular invasion; LVI, lymphovascular invasion; PNI, perineural invasion. Prefixes m and p in the stages represent MRI and pathology, respectively.

### *In Vivo* Rectal Tomoelastography in Healthy Controls and Patients

**Figure 1:**
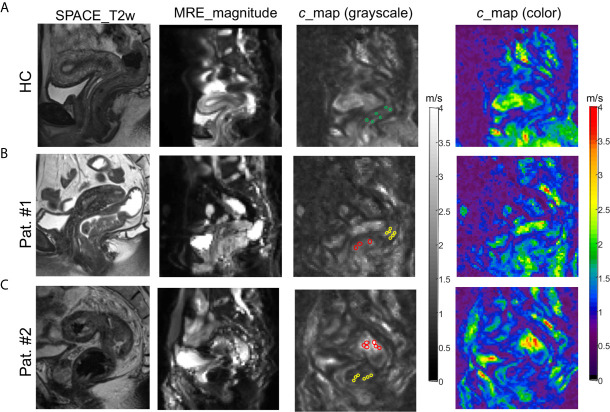
3D SPACE T2w images, MRE magnitude images, and *c*-maps (in grayscale and color) of a healthy control [**(A)**: HC] and two patients [**(B)**: Pat. #1 and **(C)**: #2] in one selected sagittal slice. The grayscale *c*-maps show the circular ROIs placed on healthy rectal wall in HC (green), distal tumor-adjacent tissue (DTT, yellow), and rectal tumor (red) in patients.

**Figure 2:**
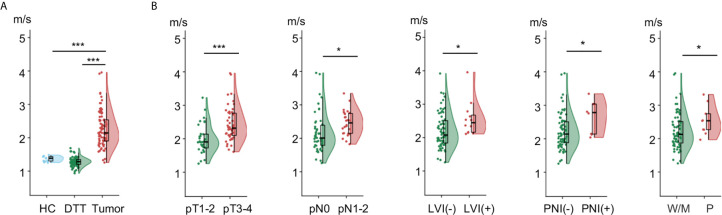
Flat-violin plot combined with boxplot of shear wave speed *c* comparing **(A)** healthy rectum in healthy control (HC), distal tumor-adjacent tissue (DTT), and rectal tumor in patients; **(B)** rectal tumor with different pathology-based pT, pN stages, LVI, PNI status, and degrees of tumor differentiation. ***p < 0.001, *p < 0.05. LVI, lymphovascular invasion; PNI, perineural invasion; -, negative; +, positive; W/M, well to moderately differentiated; P, poorly diffrentiated. Prefix *p* in the stages represents pathology.

Rectal tomoelastography procedure including the placement of surface-based drivers and 3.5 mins of continuous vibration were well tolerated by all volunteers and patients. All tomoelastography examinations were run to completion. For illustration, **Figure 1A** presents a 3D-SPACE T2w image, an MRE magnitude image, and the corresponding elastogram (*c*-map) of a healthy control (HC) in grayscale and as a color map. In HC, the rectal wall shown by the row of circular ROIs appears thin and smooth. Mean *c* in the healthy rectum was 1.4 ± 0.1 m/s. All 80 patients (mean age, 58 years± 11; 36 females) were analyzed. **Figure 1B, C** show examples of SPACE T2w images, MRE magnitude images, and the corresponding *c*-maps of one patient from the low-risk group (Pat. #1) and one patient from the high-risk group (Pat. #2). It is apparent in the *c*-maps that rectal cancer parenchyma is stiffer than the DTT and normal rectal wall in HC. As shown in **Figure 2A**, *c* in rectal adenocarcinoma was significantly higher than in DTT (p<0.0001) and healthy rectum (p<0.0001). *c* did not differ significantly between DTT and the healthy rectum.

Reproducibility was tested in HC. In all 12 volunteers (mean age, 28 years ± 10; 4 females), rectal tomoelastography was well reproducible with CR, ICC, and RADi of 0.87, 0.77 and 0.02, respectively. In a group of 15 randomly selected patients (mean age, 58 years± 10; 4 females), another radiologist independently analyzed the *c*-maps. Excellent interobserver concordance was obtained for *c* with ICC and Cronbach’s *α* of 0.958 and 0.979 for tumor and 0.777 and 0.987 for DTT, respectively.

Correlation analysis in all patients showed that *c* of rectal tumor was significantly associated with pT stage (p<0.0001), pN stage (p<0.05), degree of tumor differentiation (p<0.05) as well as LVI and PNI status (p<0.05). There was no significant correlation of *c* with sex, age, or BMI.

Furthermore, as shown in **Figure 2B**, tumors with advance pT stage, metastatic lymph node involvement, LVI, PNI, and poor differentiation status displayed significantly higher *c* values (all p<0.05). Additionally, higher *c* was also found in patients with an increased risk (p<0.001) and poorer prognosis (p<0.005). Group mean values of *c* in the different pathology-based subgroups are collected in **Table 2**.

**Table 2:** Group mean value of shear wave speed *c* in all 80 patients and collagen volume fraction (CVF) in a subgroup of 69 patients by pathology-based TN stage, LVI and PNI status, degree of tumor differentiation, prognostic stage, and risk level of all patients.

Prognostic factor	No. of patients (total n=80)	Shear wave speed *c* (m/s)	p value	No. of patients (total n=69)	CVF	p value
**pT stage**			<0.0001			0.0086
pT1-2	32	2.0 ± 0.4		27	0.13 ± 0.11	
pT3-4	48	2.4 ± 0.5		42	0.21 ± 0.12	
**pN stage**			0.013			<0.0001
pN0	54	2.2 ± 0.6		45	0.12 ± 0.091	
pN1-2	26	2.5 ± 0.4		24	0.29 ± 0.097	
**LVI**			0.017			0.1675
Negative	68	2.2 ± 0.5		57	0.17 ± 0.12	
Positive	12	2.6 ± 0.5		12	0.23 ± 0.12	
**PNI**			0.018			0.0269
Negative	72	2.2 ± 0.5		61	0.17 ± 0.12	
Positive	8	2.7 ± 0.5		8	0.27 ± 0.11	
**Degree of tumor differentiation**			0.040			0.0007
Well to moderately differentiated	71	2.2 ± 0.5		62	0.17 ± 0.11	
Poorly differentiated	9	2.6 ± 0.5		7	0.32 ± 0.084	
**Prognostic stage**			0.0022			<0.0001
Stage 0-I	26	1.9 ± 0.5		22	0.010 ± 0.082	
Stage II-III	27	2.3 ± 0.6		20	0.15 ± 0.085	
Stage III-IV	27	2.5 ± 0.4		27	0.27 ± 0.11	
**Risk stratification**			<0.001			<0.001
Low-risk	44	2.1 ± 0.5		37	0.11 ± 0.08	
High-risk	36	2.5 ± 0.5		32	0.27 ± 0.10	

CVF, collagen volume fraction; LVI, lymphovascular invasion, PNI, perineural invasion. Prefix p in the stages represents pathology.

### *Ex Vivo* Collagen Volume Fraction Quantification in Patients

**Figure 3:**
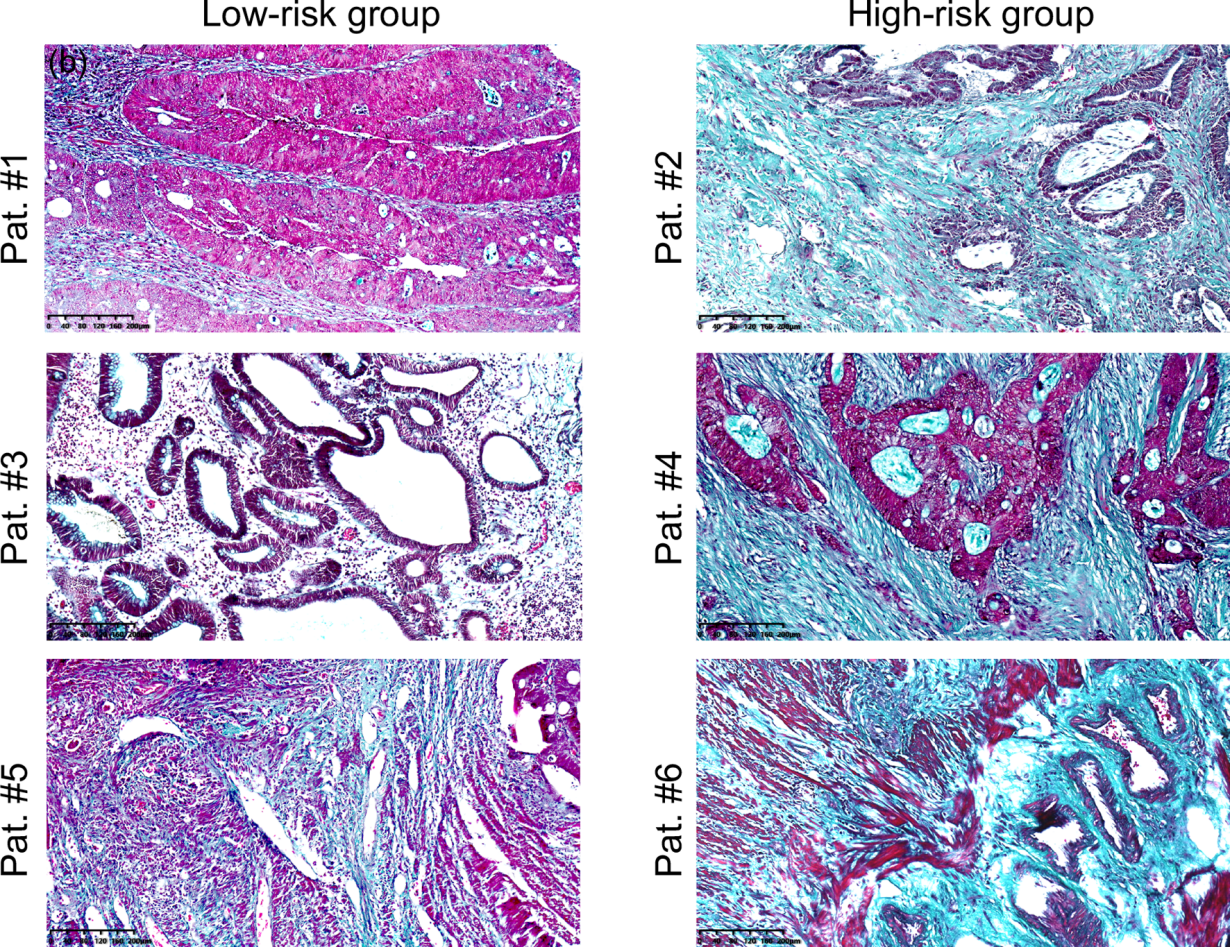
Microscopic images of rectal tumour tissues stained with Masson’s trichrome from representative patients in the low-risk (Pat. #1, #3, #5) and high-risk (Pat. #2, #4, #6) groups. Collagen fibbers was stained blue/green. Scale bars equal 40 μm. Pat.1 and Pat.2 are the same patients as shown in **Figure 1**.

**Figure 3** shows micrographs of rectal tumors stained with Masson trichrome from representative patients in the low-risk and high-risk groups as defined earlier. It was visible that compared with the low-risk group, the collagen (blue-green) content was higher and the collagen fibers were compacted to thick bundles in the high-risk group.

**Figure 4:**
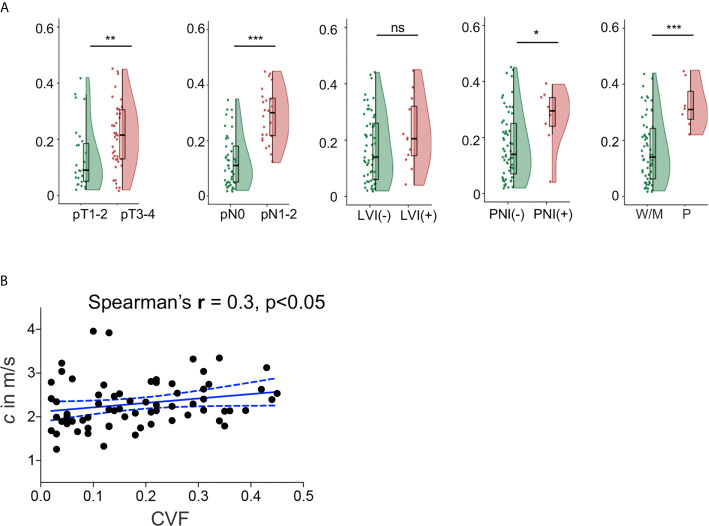
**(A)** Flat-violin plot combined with boxplot of collagen volume fraction (CVF) of rectal tumor with different pathology-based pT, pN stages, LVI, PNI status, and degrees of tumor differentiation, measured in a subgroup of 69 patients. **(B)** Correlation between shear wave speed *c* and CVF in 69 patients. ***p < 0.001, **p < 0.01, *p < 0.05. ns, no significance. LVI, lymphovascular invasion; PNI, perineural invasion; -, negative; +, positive; W/M, well to moderately differentiated; P, poorly diffrentiated. Prefix *p* in the stages represents pathology.

In all 69 patients where collagen content was quantified, similar to *c* obtained by tomoelastography, CVF was significantly higher in tumors with advanced pT and pN stages, positive PNI and poor differentiation status (all p<0.05). However, unlike *c*, no significant difference of CVF were observed between tumors with different LVI status. Significantly elevated CVF was also found in tumor samples from patients with higher risk (p<0.001) and poorer prognosis (p<0.001). Group mean values of CVF in the different pathology-based subgroups are compiled in **Table 2** and plotted in **Figure 4A**. Furthermore, correlation analysis in these patients showed that CVF of rectal tumor was positivity correlated with *c* (r = 0.3, p < 0.05), as shown in **Figure 4B**.

### Diagnostic Performance of Shear Wave Speed *c* and Comparison with MRI-Based Staging

**Figure 5:**
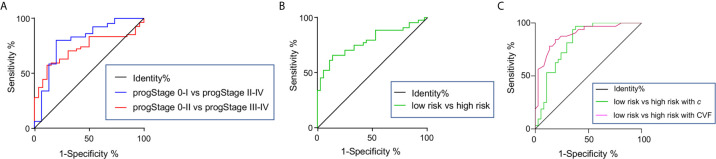
Receiver-operating characteristic curves for assessing the diagnostic accuracy of shear wave speed *c* in differentiating **(A)** pathology-based prognostic stages (progStages) and high- and low-risk levels based on pathology in the total population of 80 patients. receiver-operating characteristic curves of *c* **(B)** and CVF **(C)** in detecting high- and low-risk levels shown for a subgroup of 69 patients.

AUC for the differentiation between pathology-based pT stages, pN stages, PNI and LVI status, and degree of tumor differentiation was 0.77, 0.66, 0.72, 0.75), and 0.71, respectively. As shown in **Figure 5A**, AUC for the differentiation of pathology-based prognostic stages (progStages) 0-I *vs* II-IV and 0-II *vs* III-IV was 0.79 and 0.72, respectively. In terms of risk levels, AUC for distinguishing high-risk and low-risk patients was 0.78 (**Figure 5B**). All results pertaining to diagnostic accuracy of *c* in 80 patients are summarized in **Table 3**.

**Table 3:** Receiver-operating characteristic analysis of all 80 patients’ sheer wave speed *c* in distinguishing different pathologic categories.

Category	AUC	Cutoff (m/s)	Sensitivity (%)	Specificity (%)	p value
pT1-2 *vs* pT3-4	0.77 (0.66-0.88)	<1.9	87.23 (74.26-95.17)	60.61 (42.14-77.09)	<0.0001
pN0 *vs* pN1-2	0.66 (0.53-0.78)	<2.1	84.62 (65.13-95.64)	57.41 (43.21-70.77)	0.0232
LVI– *vs* LVI+	0.72 (0.60-0.84)	<2.9	100.00 (73.54-100.00)	51.47 (39.03-63.78)	0.0180
PNI– *vs* PNI+	0.75 (0.59-0.92)	<2.8	62.50 (24.49-91.48)	86.11 (75.94-93.13)	0.0192
W/M vs P differentiated	0.71 (0.57-0.85)	<2.1	88.89 (51.75-99.72)	56.34 (44.05-68.09)	0.0406
progStage 0-I *vs* progStage II-IV	0.79 (0.65-0.94)	<1.9	80.00 (68.23-88.90)	80.00 (51.91-95.67)	0.0004
progStage 0-II *vs* progStage III-IV	0.72 (0.61-0.83)	<2.1	57.41 (43.21-70.77)	88.46 (69.85-97.55)	0.0013
Low-risk *vs* high-risk	0.78 (0.68-0.88)	<2.1	65.91 (50.08-79.51)	86.11 (70.50-95.33)	<0.0001

LVI, lymphovascular invasion; PNI, perineural invasion; -, negative; +, positive; W/M, well to moderately; P, poorly; progStage, pathology-based prognostic stage.

In the group of 69 patients where CVF was quantified, *c* showed a very good diagnostic accuracy (AUROC: 0.82) in separating high-risk (n=32) from low-risk (n=37) patients similarly to CVF (AUROC: 0.89, p=0.32), as illustrated in **Figure 5C**.

Additionally, to assess the possible added value of tomoelastography to the standard MRI based clinical diagnostic, we compared the diagnostic performance of MRI and tomoelastography in assigning T and N stages using histopathological results as reference standard. Therefore, *c* was dichotomized using its corresponding threshold values for distinguishing pT1-2 from pT3-4 and for distinguishing pN0 from pN1-2. Based on histopathology, contingency analysis of *c*, MRI, and combined MRI and *c* yielded predictive accuracy as well as positive and negative predictive values for distinguishing pT1-2 (40) *versus* pT3-4(+) and pN0 (40) *versus* pN1-2(+). The results, summarized in **Table 4**, show that MRI was superior to *c* in differentiating pT stages (kappa: 0.92 *vs*. 0.49). Therefore, adding *c* to MRI did not improve pT staging. Since MRI and *c* had similar performance in differentiating pN stages (kappa: 0.49 *vs*. 0.38), the combination of *c* and MRI significantly improved overall pN diagnostic accuracy from 74% to 84% with a higher specificity of 83% (kappa=0.65).

**Table 4:** Contingency analysis of *c*, MRI, and combined MRI and *c* for predicting pT3-4 and pN1-2 using histopathology as reference standard.

	Overall accuracy (%)	Weighted Kappa (95%CI)	Sensitivity (%)	Specificity (%)	PPV (%)	NPV (%)
**Predicting pT1-2 (** **40** **) *vs* pT3-4(+)**						
MRI	96 (77/80)	0.92 (0.83-1.00)	98 (47/48)	94 (30/32)	96 (47/49)	97 (30/31)
*c*	76 (61/80)	0.49 (0.29-0.68)	88 (42/48)	59 (19/32)	76 (42/55)	76 (19/25)
MRI + *c*	96 (77/80)	0.92 (0.83-1.00)	98 (47/48)	94 (30/32)	96 (47/49)	97 (30/31)
**Predicting pN0 (** **40** **) *vs* pN1-2(+)**						
MRI	74 (59/80)	0.49 (0.32-0.66)	92 (24/26)	65 (35/54)	56 (24/43)	95 (35/37)
*c*	68 (54/80)	0.38 (0.21-0.56)	88 (23/26)	57 (31/54)	50 (23/46)	91 (31/34)
MRI + *c*	84 (67/80)	0.65 (0.47-0.82)	85 (22/26)	83 (45/54)	71 (22/31)	92 (45/49)

Prefix p in the stages represents pathology. PPV, positive predictive value; NPV, negative predictive value.

## Discussion

There is a need for improved staging of rectal cancer by clinical diagnostic imaging. Our study addresses this need by rectal tomoelastography which, for the first time, allowed us to quantify *in vivo* stiffness in patients with rectal cancer as a new imaging marker for ECM protein deposition. A key finding of our study was that *in vivo* stiffness correlates with the amount of collagen quantified by histopathology. Furthermore, tumor stiffness and collagen content were indicative of higher risk of aggressive rectal tumor progression that leads to a poorer prognosis.

Our data show that rectal tumor tissue is on average stiffer than DTT and healthy rectum – consistent with findings obtained *ex vivo* in colorectal cancer specimens (41). The authors of this study performed histopathological analysis and reported that elevated stiffness of rectal tumors is associated with accumulation of collagen fibers and proliferation of fibroblasts in cancer stroma (41). In our study, stiffening of rectal tumor tissue was observed from early to advanced stages, a finding that is consistent with results obtained by ultrasound-based elastography (42, 43). While these studies only examined tumor stiffness in different pT stages, our results show that rectal stiffness varies significantly not only between pT stages but also between pN stages. For this reason, tumor stiffness can be of value for differentiating pathology-based prognostic stages in rectal cancer. Moreover, our study, for the first time, shows that rectal stiffness also differentiates low-risk and high-risk patients with good accuracy. This is an important finding since risk assessment is crucial for making treatment decisions in rectal cancer. Our results show that abnormal tumor stiffness is associated with poor tumor differentiation and LVI and PNI status, suggesting that tomoelastography may be a potential marker of patient prognosis and the risk of local tumor recurrence and aggressive progression.

Histopathologic analysis revealed increasing CVF values in patients with advanced tumor pathologies. Tumor spread and invasive growth involve changes in collagen architecture which contribute largely to substantial ECM remodeling (11). For example, collagen is crosslinked and degraded in the tumor niche by enzymes such as lysyl oxidase and matrix metalloproteinases (44). In our patients, dense and bundled collagen fibers were abundantly visible in poorly differentiated rectal tumors with lymphovascular and perineural invasion. Changes in collagen content and alignment during rectal tumor progression as observed in our study could be due to the alterations of lysyl oxidase level in the neoplastic ECM which regulates collagen crosslinking as observed in CRC tissue samples (5, 6).

Earlier work on cancer biomechanics revealed that variations of biochemical and biophysical features of the tumor-hosting ECM could alter the stiffness of biological tissues across multiple tissue length scales (10, 17, 45, 46). The positive correlation between stiffness and CVF in our data suggests that tomoelastography is sensitive to alterations of ECM architecture on the microscopic level. Furthermore, our study indicates that collagen is a hallmark of advanced tumor stages and associated with the risk of aggressive progression in rectal cancer. Interestingly, tomoelastography was sensitive to lymphvascular invasion which was not detectable by CVF. This disparity in sensitivity of stiffness and CVF might be due to 1) the contribution of other ECM components than collagen such as fibronectin, proteoglycans, or glycosaminoglycans to tumor stiffness (47, 48), or 2), *in vivo* factors such as blood perfusion and vascular resistance (49) to which tomoelastography is sensitive (17). Tumor angiogenesis and the migration status of neoplastic cells into the vasculature or the lymphatic system probably better reflects lymphvascular invasion than the amount of collagen in the ECM.

Irrespective the underlying pathophysiology, our data suggested that *in vivo* tomoelastography could have important implications for the clinical diagnostics of rectal cancer. Firstly, adding stiffness as an imaging parameter to clinical MRI improved lymph node staging, which is notoriously challenging in MRI (31, 32). Conventional MRI focuses on the morphologic appearance of lymph nodes such as size and shape and is thus limited in identifying micrometastasis within the nodes. By contrast, stiffness is a biophysical parameter that scales from micro to macro, and thus allows to infer the metastatic status of a tumor from the macroscopic image contrast (50–52). Taken together, the apparent clinical impact and usefulness of rectal tomoelastography are precisely its added value to MRI for lymph node staging. Secondly, although ultrasound elastography has preliminarily demonstrated the value of stiffness for the diagnosis of rectal cancer (42, 43), unlike ultrasound-based stiffness measurements, which are performed with an endorectal transducer, tomoelastography is entirely noninvasive. Therefore, tomoelastography is better suited for screening examinations. With short acquisition times of 3.5 min, tomoelastography can easily be integrated into clinical MRI protocols. Furthermore, ultrasound elastography can only access lesions which are located within 15 cm from the anal verge while tomoelastography covers the entire colorectal segment by volumetric acquisitions.

Our study is limited by its single-center design and the lack of a large validation patient cohort. However, as this is the first application of rectal tomoelastography in patients our study was designed to demonstrate the feasibility, reproducibility, and clinical potential of this technique. Moreover, our focus at this early stage of rectal tomoelastography was on demonstrating its validity using gold-standard histopathology. Building on our encouraging results, multicenter studies with more patients and serial monitoring after treatment are planned. Tomoelastography for evaluating treatment response in patient who receive chemo-therapy and stiffness-based assessment for survival rate and cancer recurrence in a large patient cohort are planned as the next steps.

In summary, *in vivo* rectal tumor stiffness quantified by tomoelastography was positively correlated with collagen content measured by histopathology. Both markers were indicative of tumorigenic stages and the risk of aggressive tumor progression. These results suggest that collagen associated tumor stiffening due to alterations in the tumor ECM is a hallmark of rectal cancer progression and can be exploited for an improved imaging-based diagnosis, and possibly a prediction of therapeutic response.

Tomoelastography was highly reproducible and provided vital information on the tumor’s predisposition to proliferate and invade, which helped to differentiate tumors with different prognostic stages and progression risks as needed for therapeutic decision making. In term of clinical impact, rectal tomoelastography adds diagnostic value to standard MRI in the assessment of lymph nodes metastasis which is a big challenge for MRI-based clinical diagnostics in rectal cancer. Moreover, as demonstrated by this prospective pilot study, rectal tomoelastography is reproduceable, noninvasive, user-friendly and easy to incorporate into clinical imaging workflow. These features should facilitate the application of rectal tomoelastography in screening and longitudinal post-treatment monitoring. The relatively easy implementations of the technique might also promote the dissemination of tomoelastography to other clinical sites and allow conduct of larger studies in the future.

## Data Availability Statement

The original contributions presented in the study are included in the article/**Supplementary Material**. Further inquiries can be directed to the corresponding author.

## Ethics Statement

The studies involving human participants were reviewed and approved by Medical Ethics Committee of Xiangya Hospital Central South University. The patients/participants provided their written informed consent to participate in this study.

## Author Contributions

JH: Conceptualization, Methodology, Data curation, Validation, Writing- Original draft preparation, Formal analysis, Investigation. JG: Software, Methodology, Visualization, Writing- Original draft preparation. YP: Methodology, Investigation, Writing- Reviewing and Editing. PH: Software, Investigation. ML: Data curation, Validation. IS: Writing-Reviewing and Editing, WL: Project administration, Writing- Reviewing and Editing, Resources, Supervision, Funding acquisition. All authors contributed to the article and approved the submitted version.

## Funding

This work was supported by Hunan Science and Technology Department Plan Project (grant number 2018XK2304 to WL), the National Natural Science Foundation of China (grant number 82071895 to WL), and the Deutsche Forschungsgemeinschaft (BIOQIC to IS, SFB1340 Matrix-In-Vision to JG).

## Conflict of Interest

The authors declare that the research was conducted in the absence of any commercial or financial relationships that could be construed as a potential conflict of interest.

## Publisher’s Note

All claims expressed in this article are solely those of the authors and do not necessarily represent those of their affiliated organizations, or those of the publisher, the editors and the reviewers. Any product that may be evaluated in this article, or claim that may be made by its manufacturer, is not guaranteed or endorsed by the publisher.

## Supplementary Material

Supplementary Figure 1 Flowchart of patient inclusion and exclusion for MRI and tomoelastography. TRA, Transverse; SAG, sagittal; COR, coronal; LVI, lymphovascular invasion; PNI, perineural invasion. Prefix p in the stages represents pathology.
