# Supplementary figures and images for: Rectal Tumor Stiffness Quantified by In Vivo Tomoelastography and Collagen Content Estimated by Histopathology Predict Tumor Aggressiveness
Source: Front Oncol. 2021 Aug 13;11:701336. doi: 10.3389/fonc.2021.701336 (PMC8415020; doi:10.3389/fonc.2021.701336)

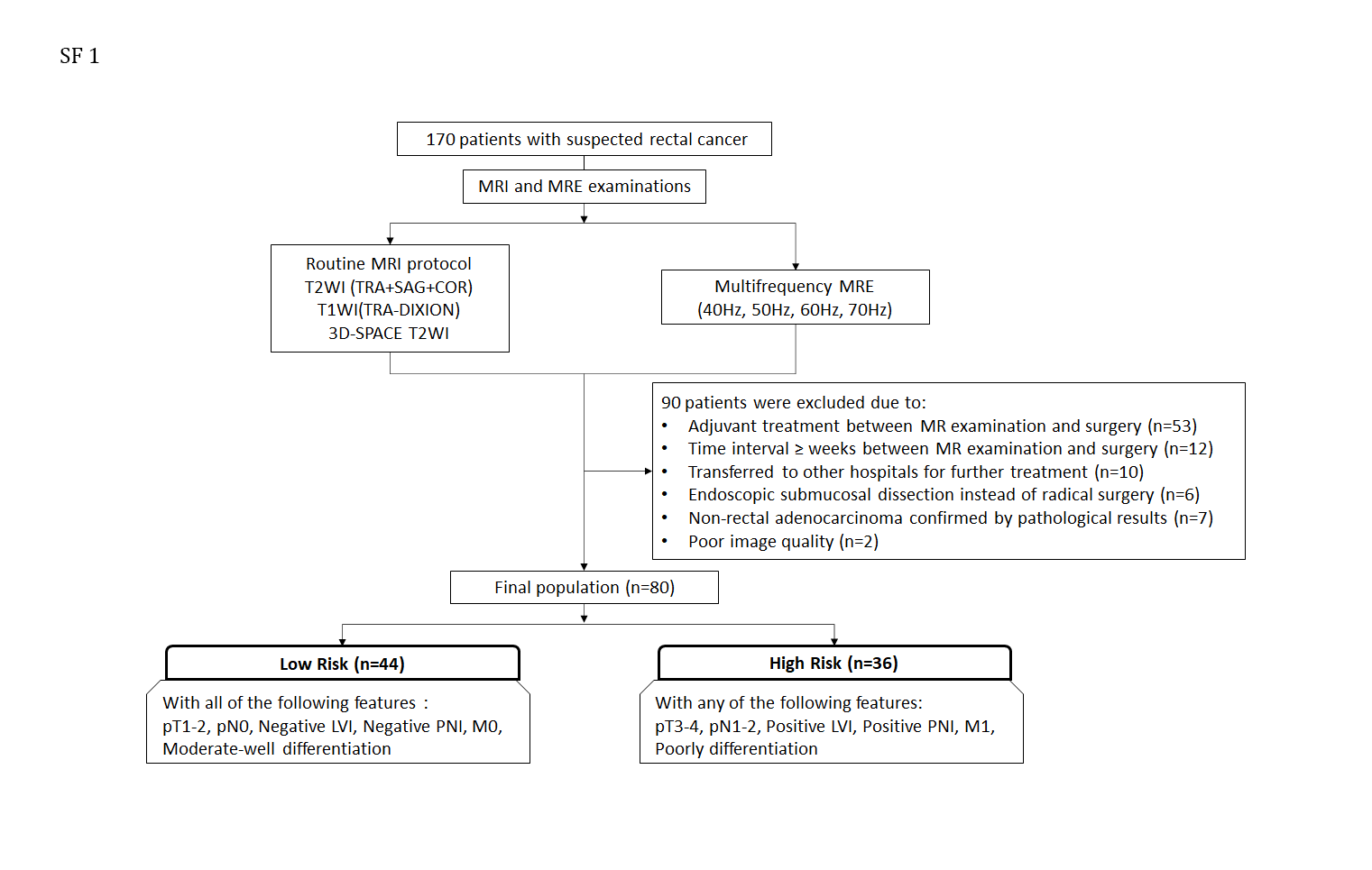

Supplement: Supplementary Figure 1 — Flowchart of patient inclusion and exclusion for MRI and tomoelastography. TRA, Transverse; SAG, sagittal; COR, coronal; LVI, lymphovascular invasion; PNI, perineural invasion. Prefix p in the stages represents pathology. [file Image_1.tif]
